# Brain Mapping in a Patient with Congenital Blindness – A Case for Multimodal Approaches

**DOI:** 10.3389/fnhum.2013.00431

**Published:** 2013-07-31

**Authors:** Jarod L. Roland, Carl D. Hacker, Jonathan D. Breshears, Charles M. Gaona, R. Edward Hogan, Harold Burton, Maurizio Corbetta, Eric C. Leuthardt

**Affiliations:** ^1^Department of Neurological Surgery, Washington University School of Medicine, St. Louis, MO, USA; ^2^Department of Biomedical Engineering, Washington University, St. Louis, MO, USA; ^3^Department of Neurological Surgery, School of Medicine, University of California San Francisco, San Francisco, CA, USA; ^4^Department of Neurology, Washington University School of Medicine, St. Louis, MO, USA; ^5^Department of Anatomy and Neurobiology, Washington University School of Medicine, St. Louis, MO, USA; ^6^Department of Mechanical Engineering and Material Sciences, Washington University, St. Louis, MO, USA; ^7^Department of Neurological Surgery, Center for Innovation in Neuroscience and Technology, Washington University School of Medicine, St. Louis, MO, USA

**Keywords:** cortical mapping, congenital blindness, electrocorticography, operative planning, functional MRI, resting-state MRI

## Abstract

Recent advances in basic neuroscience research across a wide range of methodologies have contributed significantly to our understanding of human cortical electrophysiology and functional brain imaging. Translation of this research into clinical neurosurgery has opened doors for advanced mapping of functionality that previously was prohibitively difficult, if not impossible. Here we present the case of a unique individual with congenital blindness and medically refractory epilepsy who underwent neurosurgical treatment of her seizures. Pre-operative evaluation presented the challenge of accurately and robustly mapping the cerebral cortex for an individual with a high probability of significant cortical re-organization. Additionally, a blind individual has unique priorities in one’s ability to read Braille by touch and sense the environment primarily by sound than the non-vision impaired person. For these reasons we employed additional measures to map sensory, motor, speech, language, and auditory perception by employing a number of cortical electrophysiologic mapping and functional magnetic resonance imaging methods. Our data show promising results in the application of these adjunctive methods in the pre-operative mapping of otherwise difficult to localize, and highly variable, functional cortical areas.

## Introduction

Classically described cortical mapping for neurosurgical resections primarily involves electrocortical stimulation mapping (ECS) (Ojemann et al., 1989; Berger and Ojemann, 1992) and continues to be the gold standard for intraoperative cortical mapping (Szelenyi et al., 2010). While very useful, this method has inherent limitations in the range of functions that can be mapped. This is primarily due to the requirement of overt changes in behavior or task performance to be reliably reproduced by an awake and participating subject. This may be complicated by a variety of clinical factors ranging from cognitive changes due to disease state, maturity in the pediatric population (Ojemann et al., 2003), or simply difficulty in objectively measuring changes in task performance as with memory (Perrine et al., 1994), reading (Roux et al., 2004), writing (Lubrano et al., 2004), and higher order cognitive tasks.

Individuals with pre-existing disabilities may present additional unique challenges to classical stimulation mapping. Not uncommonly patients with special needs due to physical handicap or other disability will present with neurosurgical pathology requiring intervention. When planning cortical resections for an individual with special needs, there may be added functional considerations necessary to achieve the optimal clinical outcome. Currently, most evidence-based practice derives from individuals without such disabilities. Consequently, a relative paucity of information exists when seeking treatment options, or variations to existing treatments, specially tailored to a person’s individual condition. Here the experience in tailoring cortical mapping with multiple modalities prior to surgical resection for a patient blind since the age of 3 months and seeking surgical treatment of medically refractory epilepsy is presented.

Inter-subject variability in localization of motor and speech cortex is common and needs to be individually identified to accomplish adequate resection while maximally preserving function (Ojemann, 1979). Additional cognitively important cortex may also be of particular interest in selected circumstances, but the practicality of such “tailored” mapping depends greatly on what is possible given the constraints of employed techniques. The logistics of performing a patient specific cognitive task during a mapping procedure depends heavily on whether it is intraoperative, extraoperative at the bedside, or constrained to a magnetic resonance imaging (MRI) scanner.

For tumor resections, awake craniotomies with intraoperative mapping via direct electrocortical stimulation is currently considered the optimal standard for defining the boundaries of eloquent cortex. Epilepsy surgery often differs in that a two-staged procedure involving the implantation and subsequent explantation of a subdural electrode array is used such that functional mapping occurs extraoperatively along with seizure localization. Both techniques are commonly used to identify motor and speech cortex to prevent post-operative functional deficits. Certain individuals, however, may have priorities sufficiently different from a “standard” neurologically intact patient such that deficits in functions or cognitive abilities other than those commonly mapped may result in a much more significant impact on quality of life. For a blind person there is an increased dependence on auditory and tactile sensory functions. Without sight, a blind person depends almost entirely on auditory perception to perceive and navigate the surrounding environment. Additionally, literacy is entirely dependent on a keen sense of touch when sweeping one’s fingers over a series of intricately arranged raised impressions that make up the Braille writing system.

Congenital/early blindness leads to almost certain cortical re-organization (Voss and Zatorre, 2012). For this reason in the case presented, successful identification of cortical areas involved in the functions most relevant to the patient required utilization of multiple measurement modalities. These included active task-based and resting-state functional imaging pre-operatively customized to the patient’s functional needs. During the extraoperative phase, mapping incorporated standard ECS and adjunctive techniques to record alterations in cortical physiology during different perceptual and cognitive tasks. This case study highlights the emerging capabilities of resting-state imaging and passive electrophysiologic techniques to better identify functional sites that are sensitive to cognitive operations not classically mapped and amenable to patient specific needs.

## Materials and Methods

### Patient

The patient was a 33-year-old right-handed female referred for evaluation and assessment for surgical treatment of her medically refractory epilepsy. Past medical history was significant for congenital blindness diagnosed at 3 months of age and thought to be secondary to retinal degeneration. Her initial seizure onset was at 14 years of age and subsequently became refractory to multiple medical therapies. Video-EEG (VEEG) monitoring and ictal-single-photon emission computed tomography (SPECT) imaging suggested left temporal lobe seizure onset. Subsequent PET and SPECT studies did not show consistent results convincing of a definitive pathologic focus. She was deemed a candidate for invasive monitoring for seizure focus localization and stimulation mapping. The patient continued to surgery for implant of a 48-electrode grid and adjacent electrode strips. Electrode grid and strips (PMT Medical Corp, MN, USA) were made from platinum electrodes with 4 mm diameter, 2.3 mm of exposed surface, and 1 cm inter-electrode spacing (Figure 5A). The grid configuration was a rectangular array. Clinical criterion without regard for experimental design dictated placement of all electrodes. Electrode co-registration methodology was similar to previously described methods (Hermes et al., 2010).

### Stimulation mapping

Standard ECS proceeded as determined by clinical criteria alone for delineation of the eloquent cortical areas involved in motor, sensory, and speech. Bipolar electrical cortical stimulation was delivered with a current frequency of 60 Hz and pulse width of 500 μs. Stimulation began at 1 mA at each site and ramped up to a maximum 10 mA or until after discharge threshold was reached. The sites evoking hand, tongue, mouth, and arm symptoms on stimulation ranging from 2 to 8 mA served to identify the eloquent motor and sensory cortical areas.

### Data collection

Prior to surgery the patient underwent structural and functional magnetic resonance imaging (fMRI) to assist in localizing hemispheric language dominance and areas of eloquent cortex. Structural and functional imaging acquisition followed standard methodology (He et al., 2008). The cortical surface was segmented using FreeSurfer (Dale et al., 1999) to enable registration of the imaging data on a surface rendering of the patient’s brain anatomy. Twenty-five minutes of resting-state data in addition to task performance data were acquired. Task performance included right hand motor, left hand motor, expressive language, receptive language, and passive listening tasks. The language tasks were based on those of Gaillard et al. (2007) and customized to the patient’s performance abilities. Auditory cues prompted pressing a fiber optic button with the right or left hand index finger for a motor task. All fMRI runs were completed in 10 blocks of control and 10 blocks of task continued up to 100 blocks. Imaging repetition times were 2.5 and 3 s for motor and language tasks, respectively. The blood oxygen level-dependent (BOLD) signal changes were then used to identify the hand area of primary motor strips bilaterally and hemispheric dominance in language. As previously described (Gaillard, 2004), BOLD signals were calculated in a voxel-by-voxel difference analysis from task versus rest epochs. Statistical *t*-maps were generated and used for interpretation of results.

Functional connectivity maps of relevant cortical resting-state networks (RSNs) were generated from at rest, spontaneous BOLD signals. Voxel-wise RSN membership using a novel multi-layer artificial neural network (ANN) classifier was computed (Hacker et al., 2013). This algorithm first computes resting-state correlation maps for each voxel, and then computes network membership by evaluating weighted combinations of spatial features extracted from that voxel’s map that are consistent with features learned about RSNs during a training process. Training data consisted of a set of correlation maps generated with seed ROIs from a task meta-analysis (dorsal attention, ventral attention, motor, visual, fronto-parietal control, language, default mode) for each of 21 young normal control subjects. After training the network using the back-propagation algorithm (Rumelhart et al., 1986) to classify these images (100% training accuracy), classifier performance was validated on an independent 17-subject test set (99.6% test accuracy). The classifier delivers seven maps, one for each network (Lee et al., 2012). In this manuscript we show only the voxel-wise estimates for the language and motor network.

The electrodes were co-registered with pre-operative T1 MPRAGE anatomic images in a method similar to that previously described by Hermes et al. (2010). CT scans were acquired after grid implant and aligned to pre-operative MR imaging. CTs were transformed to atlas space using a cross-modal procedure based on alignment of image gradients (Rowland et al., 2005). Electrodes were segmented by a combination of voxel erosion, a 5 mm Gaussian blur, and thresholding. Center-of-mass coordinates from clusters of face-contiguous voxels were then isolated. The electrodes locations at the time of CT acquisition are generally displaced inward relative to the location of the cortical surface at the time of MRI acquisition due to grid implantation. To correct for this, electrode coordinates were projected to the surface of the brain along a path path normal to the surface of the grid. The surface anatomy used in this procedure was extracted using Freesurfer 5; the segmented pial surface was filled and then blurred modestly (2 mm) such that electrodes arrive at a location reflecting the smoothed convexity of the brain. After comparing distributions of inter-electrode distances before and after this procedure, we estimate the total amount of error introduced by our localization procedure to be 2–3 mm. This is small compared to the smoothing performed in fMRI analysis (6 mm Gaussian kernel) and in agreement with the results of Hermes et al. (2010) (2.4 ± 0.8 mm).

The patient underwent continuous clinical monitoring in the Epilepsy Monitoring Unit. Experiments were conducted at the patient’s bedside via splitter-cable allowing ECoG data collection simultaneous to clinical monitoring. Analog signals were digitized by FDA approved g.Tec USB biosignal amplifiers (Guger Technologies, Graz, Austria) and stored on a Dell computer (Dell, Houston, TX, USA). The general-purpose brain-computer interface system BCI2000 (Schalk et al., 2004) was used for time-locked stimulus presentation and data recording. All signals were sampled at 9600 Hz for adequate speech recording from time-locked microphone data and then down-sampled to 1200 Hz for digital signal analysis.

### Task design

A battery of experimental tasks was designed to localize functions of clinical importance to the patient. In addition to mapping classic regions of motor and speech, cortical areas associated with perceiving environmental sounds and Braille reading were tested and identified.

First, in a simple word repetition task, presented in a block-design paradigm, the patient heard and repeated once monosyllabic words by speaking into a microphone. Recorded microphone signal data was time-locked to the ECoG signal by BCI2000. The word list for this task was as follows: beat, heed, keen, lead, mean, peep, read, seat, teen, bet, head, ken, led, men, pep, red, set, ten, bat, had, can, lad, man, pap, rad, sat, tan, boot, hood, coon, lewd, moon, poop, rood, soot, toon.

In a similar design, a second concept association task was performed. Instead of simply repeating a monosyllabic word, a different word with emotional context was presented and the patient was instructed to generate a conceptually related word again spoken into the microphone. The word list for this task was as follows: joy, frailty, calm, courage, energy, despair, tiredness, maturity, peace, stupidity, shock, brutality, disbelief, relaxation, honesty, worry, curiosity, wit, determination, jealousy, delight, graciousness, sadness, pain, loyalty, dexterity, fear, fragility, wisdom, disappointment.

Third, a Braille reading task was performed in a similar block-design paradigm where an audible cue was presented at the beginning of a trial instructing the patient to begin reading Braille continuously from a book. A similar audible stop cue instructed the patient to stop and rest at the end of the trial. For all tasks the patient was instructed to rest calmly during the inter-trial intervals (ITI) until the next trial period began. Trial periods were 4, 6, and 10 s, with ITI of 2, 2, and 5 s, for the word repetition, concept association, and Braille reading tasks, respectively. Trial repetitions varied depending on patient fatigue from task performance as well as the various factors related to the post-operative inpatient hospital setting. Total trial repetitions were *N* = 195, *N* = 30, and *N* = 30, for the word repetition, concept association, and Braille reading tasks, respectively.

A quasi real-time method of brain mapping was also used. The SIGnal modeling For Real-time Identification and Event Detection (SIGFRIED) package for BCI2000 enables real-time presentation of results that converge to a stable solution in a relatively short period of time. This provides a quick and efficient method of mapping similar functions without the need for a block-design task structure (Schalk et al., 2008a,b). SIGFRIED has successfully been applied in the extraoperative (Brunner et al., 2009) as well as intraoperative (Wu et al., 2010) settings. Here we chose four tasks for real-time mapping: (1) reading Braille, where an auditory cue signaled when to actively begin and end reading continuous Braille text; (2) environmental sounds, where environmental sounds were passively attended via headphones; (3) speech sounds, where the English language speech was heard via headphones; (4) speaking, where an auditory cue signaled when to recite the pledge of allegiance. Each task was presented in sequential trials for 1.5 s, with 1.5 s ITI. The sequence of four task trials was then repeated five times.

### Analysis

The word repetition and concept association tasks were segmented into a listening period, during which the patient heard the presented word, a speaking period, during which the patient spoke a word into the microphone, and rest periods consisting of the preceding ITI. The listening period onset was marked by BCI2000 corresponding to the onset of audible word presentation. The speaking period was defined as 250 ms prior to voice onset time. Voice onset time was identified programmatically by an increase immediately following the stimulus period in the power envelope of the microphone data channel that had been band-filtered at 100–300 Hz to reduce line noise and preserve spoken voice. Braille reading and rest periods were marked by BCI2000 corresponding to the presentation of start and stop cues, respectively.

Electrocorticographic frequency alteration mapping (EFAM) analysis was applied to block-design task data resulting in statistical measures of activation defined by modulations in frequency band power at individual electrode sites. The power-spectral density (PSD) from equal length representative samples of each active period (i.e., hearing, speaking, or reading) and rest period for each task was obtained in 1 Hz bins using a 250 ms Hanning window with 50% overlap. For each frequency bin, the correlation coefficient (*r*^2^) between active periods and rest periods was calculated to detect a change in spectral power associated with task performance. A *t*-statistic and corresponding *p*-value were then calculated and a *p*-value of<0.05 accepted as statistical significance for a given frequency bin. A 75–115 Hz frequency band, which is in the Gamma range, was used to identify cortical sites active with task performance. An active site was defined as having at least one bin in the range with a statistically significant increase in power (Leuthardt et al., 2007; Miller et al., 2007b; Wu et al., 2010; Roland et al., 2011). The maximum *r*^2^ value for each electrode was then used to plot a topographical activation map on a polygonal mesh of the patient’s brain (Figure 3).

Signal modeling for real-time identification and event detection analysis was performed as previously described (Schalk et al., 2008b). The high-gamma frequency range of 75–115 Hz was selected for real-time analysis by the SIGFRIED toolkit. The SIGFRIED algorithm, described previously in greater detail (Schalk et al., 2008b; Brunner et al., 2009), provides a real-time metric for identifying cortical areas of activation. This is achieved by first sampling resting-state ECoG data and developing a multiple Gaussian distribution model of the data. This model is then compared against task data and a Mahalanobis distance is calculated between new samples and the multiple Gaussian distributions to provide a metric of similarity to resting state. The inverse of this similarity to baseline is then scaled and visually presented as enlarging circles corresponding to respective electrode locations, which represents activations relative to baseline associated with the task being performed.

## Results

### Clinical assessment

Clinical assessment included standard VEEG monitoring, pre and post-operative neuropsychiatric testing, and diagnostic SPECT and MR imaging. Wada testing was not performed per our standard clinical practice in right-handed patients. VEEG monitoring successfully captured multiple clinical seizure events with correlated electrographic abnormalities. EEG recordings were most suggestive of left temporal seizure onset.

Structural MRI repeatedly suggested decreased size of the left hippocampus without evidence of underlying architecture disturbance. Mesial temporal sclerosis was not appreciated on pre-operative imaging. MR spectroscopy measured decreased levels of *N*-acetylaspartate throughout the left hippocampal head, body, and tail. Additionally, ictal and interictal SPECT imaging was most consistent with a seizure focus within the left temporal lobe.

Neuropsychiatric assessment found the patient to demonstrate high average estimated intellectual function with intact cognition and no clearly lateralizing or localizing findings. At that time she was also suffering from mild depression.

### Functional MRI

**Figure 1:**
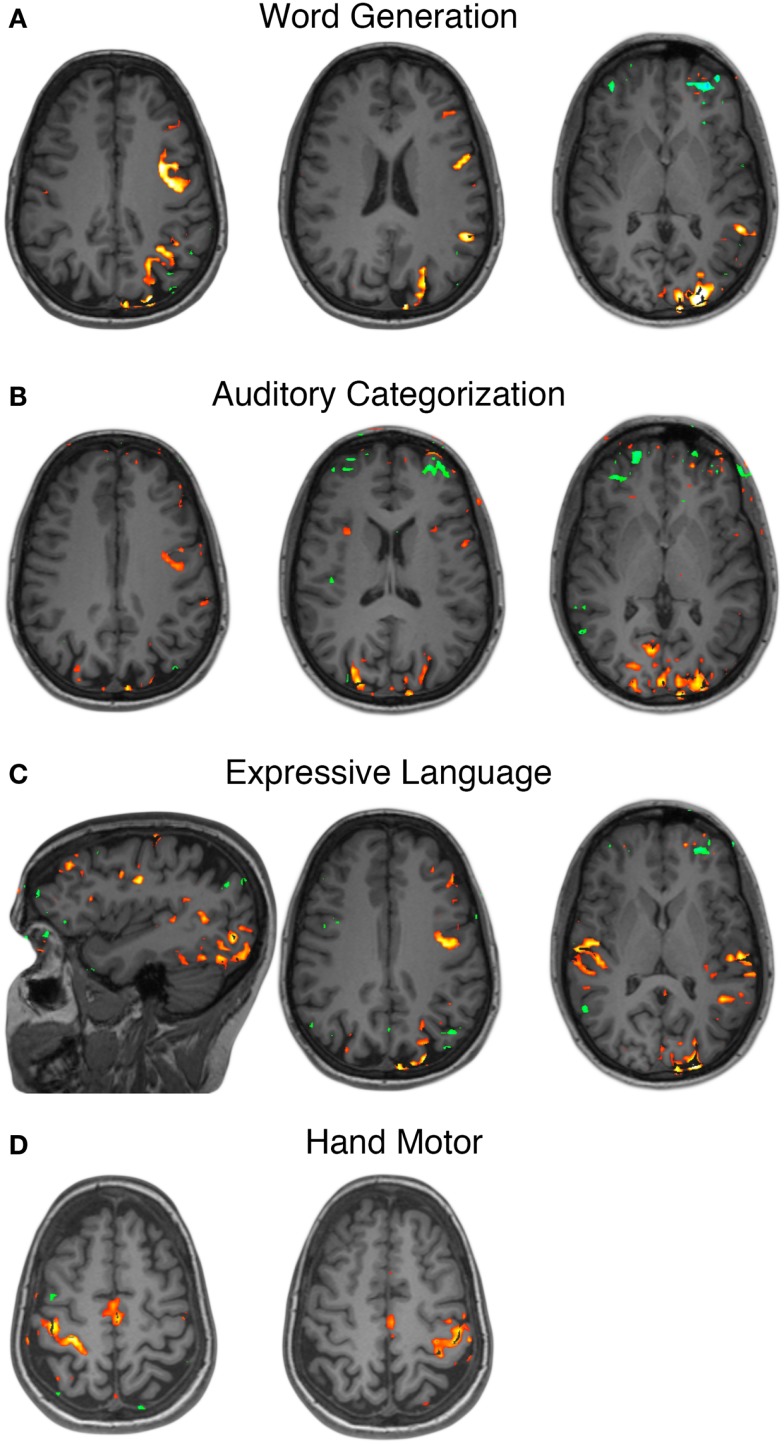
**Functional MRI localization of speech and motor cortex**. Functional MRI for various language tasks revealed left sided dominance as well as recruitment of posterior occipital lobe cortical areas **(A–C)**. Unilateral hand motor tasks revealed primary motor cortex activations in the contralateral pre-central gyrus **(D)**.

Pre-operative fMRI testing was performed with simple motor tasks and an array of speech tasks designed to emphasize various components of the speech network. All language tasks demonstrated strong left hemisphere dominance in BOLD signal activations. In addition to BOLD signal activations in the classic peri-sylvian regions of posterior-inferior frontal lobe and posterior-superior temporal lobe, there was also extensive recruitment of the occipital cortex, greater on the left than the right, across all language tasks (Figures 1A–C). The affected cortex included calcarine sulcus, cuneus, lingual, lateral occipital, etc. Unilateral simple hand motor tasks elicited BOLD signal activations synchronized with the paradigm and localized to the contralateral pre-central gyrus (Figure 1D). These activations corresponded to the Omega sign representing the hand area of the motor homunculus (Yousry et al., 1997).

### Resting-state functional connectivity MRI

**Figure 2:**
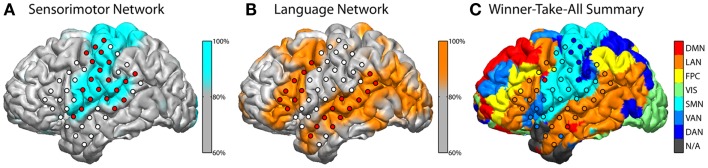
**Classification of resting-state fcMRI networks**. Classifier output for the sensorimotor network (SMN) and language network (LAN) are shown above in blue **(A)** and orange **(B)**, respectively. Values were rescaled to a percentile of across all voxels and a threshold at the 80th percentile is illustrated after interpolating to the reconstructed anatomy. Classifier values were sampled to electrodes, which are filled with red to indicate that nearby (up to 5 mm) gray matter voxels were, on average, in the 80th percentile or higher. White electrodes fell below this threshold. A winner-take-all (WTA) summary across all networks **(C)** was computed and displayed in similar fashion with respective coloring. Each voxel was assigned a color based on the network with the highest class-score. The resulting map was interpolated to the subject’s surface anatomy and to each electrode. The networks illustrated include the default mode (DMN), language (LAN), fronto-parietal control (FPC), visual (VIS), somatomotor network (SMN), ventral attention (VAN), and dorsal attention (DAN). N/A indicates that no network achieved a classifier score above threshold.

The subject exhibited grossly normal RSN topography in most connectivity networks, though some differences were notable. The extent of the language network (Figure 2B) was broader than in normally sighted individuals (Lee et al., 2012; Hacker et al., 2013). This was notable for the posterior language area, which extended across the angular gyrus, which is an area normally associated with the default mode network (Raichle and Snyder, 2007). The language area also appreciably extended further into the occipital lobe. The fact that the RSN classifier identified a “visual” network (labeled green in Figure 2C) may first seem counter intuitive, however this may be suggestive of cortical plasticity. The RSN classifier identifies voxels belonging to correlation maps that are spatially similar to pre-defined RSN distributions, which are previously defined during training. In the present case, these voxels still exhibited a pattern of connectivity commensurate with a canonical visual network and were therefore labeled accordingly. However, the task-based fMRI results in this subject revealed regions, which would classically be considered part of the “visual” system, that here were found to be activated by auditory and language tasks.

### Electrocorticographic frequency alteration mapping

**Figure 3:**
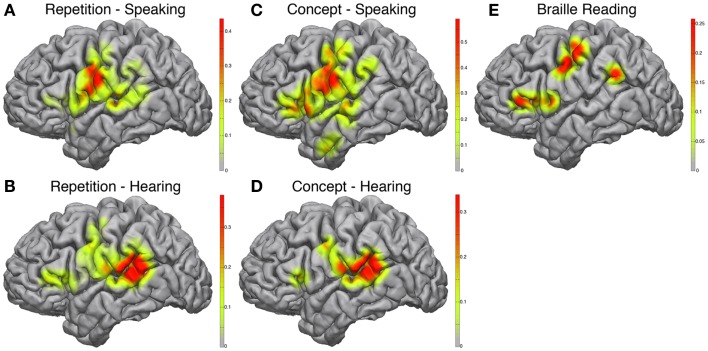
**Electrocorticographic frequency alteration mapping**. Electrocorticographic frequency alteration mapping is shown for the repetition **(A,B)** and concept association **(C,D)** tasks. Activations were seen in the ventral peri-Rolandic and posterior-ventral frontal lobe during speaking epochs **(A,C)** and in the posterior-superior temporal gyrus during hearing epochs **(B,D)**. A block-design Braille reading task was also performed revealing cortical activations in the peri-Rolandic convexity consistent with hand area of the homunculus and in the supramarginal gyrus and posterior-ventral frontal lobe **(E)**.

During the speaking portions of the Word Repetition and Concept Association tasks, the posterior-inferior frontal cortex approximating mouth motor cortex was activated in both paradigms (Figures 3A,C), with a greater correlation in the Concept Association task. In addition, we observed a stronger activation in the posterior-inferior frontal gyrus, just anterior to the motor strip, approximating Broca’s area in the Concept Association task compared to the Repetition task. We used two different speech paradigms to attempt utilization of more of the speech network. The complexity of speech production and the cortical elements contributing to the various forms of speech are actively being researched and beyond the scope of this study (Blank et al., 2002). However, it is of relevance to note that there is evidence of that propositional and non-propositional speech may dissociate (Speedie et al., 1993) and without an exhaustive experimental mapping cortical areas contributing to forms of speech may not be readily identified.

The hearing components of each paradigm demonstrated very similar maps (Figures 3B,D) with strong activations located in the posterior-superior temporal gyrus, near the temporo-parietal junction, approximating classical Wernicke’s area. Again this is not unexpected, as during this part of the task paradigm receptive areas of the speech network have been shown to be the most active (Towle et al., 2008).

Braille reading demonstrated cortical activations that incorporated both somatomotor and speech related cortex (Figure 3E). In accordance with the dependence of Braille reading on digital sensory input, peri-Rolandic activations in proximity of hand motor-sensory cortex showed strong activations during active Braille reading. Additional activations were seen in the posterior-inferior frontal gyrus, similar to those seen during the speaking component of the Concept Association paradigm, as well as at the temporo-parietal junction.

### Signal modeling for real-time identification and event detection

**Figure 4:**
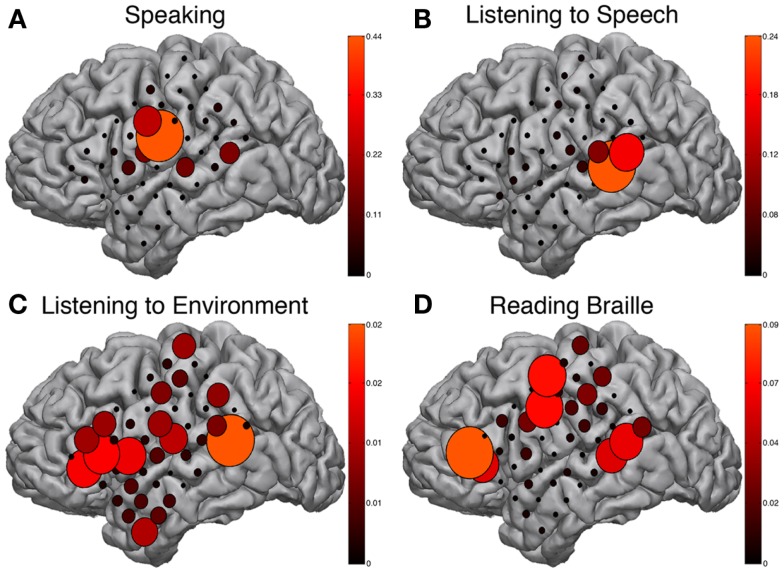
**SIGFRIED mapping**. Results from SIGFRIED mapping of speaking **(A)**, passive listening to speech **(B)**, passive listening to environmental sounds **(C)**, and continuously reading Braille **(D)**. Size and color hue of circles are linearly related and represent correlation of signal changes with task performance from baseline signal profile recorded during rest. Each sub-figure scaled independently to max SIGFRIED score for respective task.

The BCI2000 real-time mapping software suite SIGFRIED was used to augment the EFAM findings. Tasks such as reading Braille and listening to speech or environmental sounds may be resistant to traditional block paradigm task designs and therefore could benefit from the advantages of real-time activation mapping provided by SIGFRIED. The speaking task, during which a familiar internally generated speech was repeated continuously, showed strong activations in the ventral peri-Rolandic area corresponding to mouth sensory-motor cortex (Figure 4A). These activations were congruent to known expressive speech networks and correspondingly activated during speaking periods of both block-design EFAM paradigms.

Receptive speech was tested while passively listening to English language speech and showed strong activations in the posterior-superior temporal gyrus (Figure 4B), a region approximating classical Wernicke’s area. These activations were in known receptive speech networks and were similarly activated during speaking periods of both block-design EFAM paradigms.

Passive listening to environmental sounds was also tested but did not show strong consistently correlated activations in a meaningful distribution (Figure 4C). The most responsive site occupied the posterior-superior temporal gyrus, which is an area that previous results implicate in receptive speech comprehension and may therefore be part of auditory associative networks (Edwards et al., 2005).

Braille reading maps also showed reliable areas of activation suggestive of both sensorimotor and speech network. Activations clustered around three areas corresponding to peri-Rolandic hand sensory-motor cortex, posterior-superior temporal gyrus approximating Wernicke’s area, and posterior-inferior frontal gyrus approximating Broca’s area (Figure 4D). These areas were comparable to those demonstrated by EFAM during Braille reading in a more structured block-design task.

### Stimulation mapping

**Figure 5:**
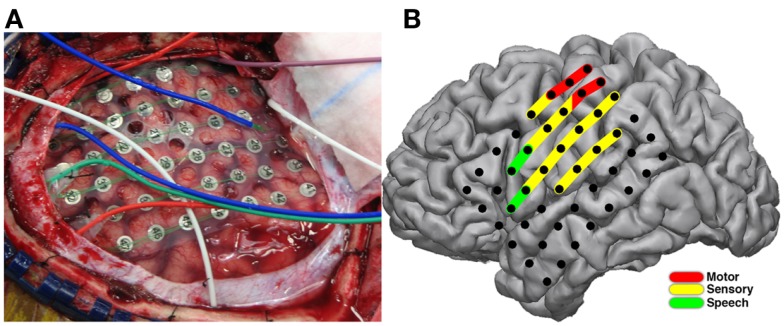
**Electrode placement and results of stimulation mapping**. Placement of ECoG electrodes **(A)** for invasive monitoring and localization of epileptic foci are seen during the initial operative procedure. Results from extraoperative bedside bipolar electrocortical stimulation mapping **(B)** showing induction of speech arrest (green) in classical Broca’s area, and sensory perception (yellow) and motor effects (red) in peri-Rolandic areas.

Stimulation mapping was performed at the patient’s bedside without complication. Speech arrest was noted at two sites in classical Broca’s location with stimulation current ranging from 4 to 8 mA. Motor and sensory changes were found along peri-Rolandic electrodes (Figure 5B) with stimulation current ranging from 2 to 10 mA. After-discharges were noted at currents ranging from 4 to 10 mA.

### Operative outcome

Invasive mapping of the patient’s epileptic foci successfully recorded multiple typical seizures suggesting origination in the mesial temporal lobe. Given these results and the information from stimulation and ECoG mapping, the patient elected to continue to the operating room for left sided craniotomy and selective amygdalohippocampectomy (AHC). The surgical corridor to pursue a selective AHC was defined by our adjunctive mapping results in order to provide the patient with the greatest chance of preserving additional speech function and sensory perception. Specifically, signs of cortical activation during speech and environment listening paradigms were found in the anterior temporal lobe. These are evident in Figure 3C and Figure 4C of the interactive EFAM and SIGFRIED mapping results, respectively. The resting-state speech network also included much of the temporal lobe including a portion of the anterior temporal lobe as noted in Figure 2B of the functional connectivity MRI (fcMRI) data. A selective AHC through the mid portion of the middle temporal gyrus, somewhat more posterior than the usual approach, was then elected because it was thought to avoid these regions of functional importance. The conjunction of these findings provided a very tailored assessment and modified surgical approach that enabled her to undergo surgery without complication or event. Pathologic analysis of hippocampus specimens revealed neuronal loss and mild bland astrocytosis within the end folium of area CA4. These findings were consistent with hippocampal sclerosis. On post-operative outpatient follow-up she continued on her anti-epileptic regimen of Levetiracetam and Lamotrigine and reported significant improvement in her seizures.

At 2 years post-operative follow-up the patient has continued to be seizure free, representing an Engel class 1 outcome. She has begun antidepressant medication and shown an improvement in her mood. No changes to her AED medications have been made thus far and she continues to follow with her primary epileptologist. The patient returned for repeat neuropsychiatric evaluation approximately 1 year after surgical resection. Her chief complaints were of decreased memory function since surgery. Objective testing suggested a moderate deficit in verbal memory and a mild deficit in auditory naming. No new deficits in speech or Braille reading were reported. Cognition remained otherwise intact.

## Discussion

In this study, numerous brain mapping approaches evaluated functional regions in a patient with congenital blindness. The case highlights the importance of using complementary techniques to identify classic and non-classic eloquent cortex. Notably, passive mapping techniques such as fMRI and passive electrocorticographic recordings enabled identification of sites associated with auditory perception and Braille reading, which would be extremely difficult to localize with classic stimulation paradigms. We describe our electrophysiological mapping as “passive” to differentiate from the canonical stimulation based mapping methods that insert current into the brain for a functional effect. It should be noted that the techniques described here still require cooperation with the patient and participation in the task paradigm. While there are still challenges that may complicate patient cooperation, particularly in a recently post-operative patient, the ability to minimize cortical stimulation and associated morbidities is the aim of adjunctive measures demonstrated in this study. In addition to the clinical relevance of these mapping findings, the cortical networks and activations associated with various cognitive paradigms revealed how congenital blindness affected the task-based functional architecture but not the resting-state connectivity of the brain.

### Clinical implications

High quality, invasive, cerebral cortical mapping continues to be a surgical necessity. The importance of such mapping is well established in the neurosurgical literature (Ojemann et al., 1989; Berger and Ojemann, 1992; Duffau et al., 2005). Direct electrical cortical stimulation for the induction of temporary cortical lesions is the currently accepted standard for identifying regions essential for somatomotor or speech function (Szelenyi et al., 2010). Identification of these functional sites is accomplished by locally passing current through the brain to cause the arrest or alteration of the cognitive operation of interest. This inherently invasive approach requires direct application of an electrical stimulus to the cortical surface. Induction of seizures is a risk of stimulation mapping that depends on a careful balance of stimulation level necessary to arrest function without causing significant after-discharges or generalized seizure activity (Szelenyi et al., 2007). This is a known complication inherent to the technique. Neurosurgeons and epileptologists experienced with stimulation mapping are able to minimize this risk, but not completely eliminate seizure induction or its related complications (Sartorius and Berger, 1998). While stimulation mapping does have inherent morbidities that may challenge or even completely prevent achieving reliable results, it also maintains certain advantages when successful that make it clinically essential. The interpretation of stimulation results as described by the patient and observed by a well-trained epileptologist may provide insights unparalleled by objective techniques. Examples of clinical interpretation of events such as hallucinatory perception or other distortions of perception during cortical mapping are present in the literature and may be clinically relevant to patients (Blanke et al., 2002; Parvizi et al., 2012).

In addition to the clinical risk of seizures, another important limitation to stimulation mapping is the need to monitor a cognitive operation (speaking and moving) and objectively identify arrest or alteration of function. For simple motor or speech tasks, an objective third party observing the patient can verify the results of stimulation. Sensory perception such as hearing, however, is more difficult to validate objectively through cortical stimulation. Stimulation induced alteration depends on the subjective reporting by the patient and cannot be externally verified. In this case, cognitive operations such as Braille reading and hearing are of increased importance to a blind person who depends exclusively on these capabilities. Here the use of passive recording techniques such as EFAM and SIGFRIED provided objective measures of cortical activations associated with these cognitive operations. Whether presented in a block design or in quasi real-time, these measures can be more readily customized to the patient’s cognitive needs than is possible for stimulation paradigms that require observer input.

There are important strengths and weaknesses of the different passive recording and signal analysis designs. The clinical mapping technique known as EFAM (Leuthardt et al., 2007; Wu et al., 2010) identifies cortical activations manifested in spectral power changes, associated with performance of a given task by the respective individual. EFAM employs a block design in which the task alternates with rest and involves statistical assessment of consistent changes in both low (8–32 Hz) and high frequency (75–100 Hz) power. The crucial benefit of EFAM is the broader spectrum of tasks that can be reliably and rigorously mapped as compared to ECS. However, EFAM requires persistent participation and attention throughout the task, which can sometimes be difficult for an invasively monitored subject. A repetitive block-design paradigm using the SIGFRIED real-time mapping software suite for the BCI2000 framework (Schalk et al., 2008b; Brunner et al., 2009) addresses this limitation. The benefit of such a real-time mapping technique is in minimizing the confounding factor of compliance with task performance. In post-operative patients who have electrode arrays implanted, concentration and task fatigue are significant factors limiting reliable data collection. This is also true when performing mapping during awake craniotomy procedures (Wu et al., 2010). Thus, reducing the time required to gather reliable data is of critical importance. Methodological counterparts described in the literature for real-time mapping have been shown to produce similar results under different analytic procedures (Miller et al., 2007a). In this case, both methods produce compelling and consistent results in identifying locations associated with vital patient specific cognitive operations that proved clinically informative. Specifically, while ECS was successful in localizing sensorimotor and speech sites, they were only localized to the frontal lobe (Figure 5B). The passive recording techniques provided additional data for perceptual tasks that localized activation in the temporal lobe, a clinically significant locus given that seizure onset in the patient involved mesial temporal structures.

A complement to invasive mapping is BOLD fMRI, a readily accessible means to achieve initial mapping data for pre-operative planning. As seen in Figure 1, however, precise localization to the degree of accuracy desired for surgical guidance was not achieved and was confounded by co-activated regions not “essential” for the given task. This can represent an inherent limitation of non-lesional methods (i.e., not using electrocortical stimulation) such as fMRI, electrocortical mapping, and other passive techniques. A more recent fMRI mapping technology that can reduce the risk associated with coactivation is determining resting-state fcMRI for pre-defined networks. While a relatively new technology, currently in its infancy of translation to clinical practice, fcMRI has the advantage of passively identifying sites involved in selected cortical networks (Shimony et al., 2009; Zhang et al., 2009). One advantage to passive methods is to avoid the reliance on reliably reproducible block-designed task performance. Instead, cortical networks known to involve, for example, primary motor, speech, frontal eye fields, etc., are mapped based on the correlation patterns of low frequency BOLD signal fluctuations during the resting state (i.e., not associated with task performance). The data presented in this case showed utility in identifying the re-organization of speech networks to classically visual cortical areas. Similarly, areas known to be involved in vision and gaze were lacking in these data from a congenitally blind individual. Plastic re-organization was predictable in such a case and here shown to be identifiable by passive imaging methods without the need for a repetitively reproducible block-design task.

### Scientific relevance and relation to previous literature

Prior research on altered localization of cortical function in blind individuals has especially relied on functional MRI (Burton et al., 2002; Sadato et al., 2002; Burton, 2003). Acquiring direct electrophysiologic data (e.g., EFAM or SIGFRIED) on these types of patients is only possible due to the unusual circumstance of a blind individual requiring invasive monitoring. The re-organization that occurs in blind individuals has been previously described (Voss and Zatorre, 2012). Prior fMRI research has consistently shown visual cortical recruitment for language tasks. This has been reproduced in early and late onset blinded individuals to further characterize the subcomponents of language represented in visual cortical areas (Burton et al., 2003). A unique aspect of language function in the occipital cortex of vision impaired concerns the importance of semantic aspects of language, whether obtained through reading Braille (Burton et al., 2002) or categorizing heard words (Burton, 2003; Burton et al., 2003). Similar visual cortical activations during a non-language, tactile discrimination task have been shown and further characterized among early blind, late blind, and sighted individuals, with gradation of visual cortical recruitment respectively (Burton et al., 2004).

The combined imaging and electrophysiologic data complement these previous findings. The task-based fMRI data certainly provided evidence for recruitment of occipital regions (Figure 1A). When looking at the RSNs, there was also evidence that the language network extended into the occipital lobe and lateral/basal temporal lobe more substantially (Figure 2). There was, however, still a separable occipital polar network, putatively a separate “visual” network. These findings potentially imply that plasticity can occur within the fundamental architecture of resting-state networks. Consequently, while networks may change regarding functions, they cannot be fully co-opted. Also interesting were the similarities and differences seen between activations to spoken language and Braille reading. As mentioned, prior studies have shown that Braille reading associated with activations in visual areas, supporting the notion that these sites have been adapted to a novel language function. When comparing the cortical activation of Braille reading (a language task) to that of auditory speech processing (also a language task), the cortical maps have relatively distinct topographies. These findings are supported by prior research which has shown separable activations associated with the two language tasks. Braille reading of continuous text required syntactical parsing and lexical memory to comprehend Braille rendered text. This required memory, semantics, and syntax when engaging occipital cortex in the congenital blind patient. One sees the same regions activated in early blind subjects with semantic tasks delivered through listening to words (Burton, 2003; Burton et al., 2003). Less cognitively demanding language tasks, like rhyming (Burton et al., 2003) or the word repetition task used in the current study activated lesser extents of occipital cortex. Emmorey et al. (2007) identified the supramarginal gyrus and superior parietal lobule to be more active when reading Braille. This is in accordance with our finding of supramarginal gyrus activation with Braille reading (Figure 3E) but not with spoken repetition of heard words (Figures 3A,C). The activation of the inferior parietal supramarginal gyrus and also occipital cortex in early blind (Burton et al., 2012) are consistent with the processes of focused attention on tactile inputs when reading Braille and attention to memory for the lexical content of the text. Further, this distinction would support that, while Braille reading and repetition of heard words similarly related to language, these tasks were subserved by distinct cortical populations much in the way that speech from different languages have different cortical topographies (Kim et al., 1997).

## Conclusion

In the challenging situation where cortical re-organization is present and identifying atypical cognitive operations is clinically important (such as auditory function and Braille reading in the blind), mapping techniques less reliant on cortical stimulation and observing alteration of function are important. Using an integrated approach of resting-state fcMRI and passive cortical physiologic methods, such as EFAM and SIGFRIED, may provide additional insight into the cortical architecture and activations that would be very difficult to localize with standard stimulation paradigms.

## Conflict of Interest Statement

Eric C. Leuthardt holds stock in Neurolutions, General Sensing, and OsteoVantage. The other co-authors declare that the research was conducted in the absence of any commercial or financial relationships that could be construed as a potential conflict of interest.
